# Mapping Neuroscience in the Field of Education through a Bibliometric Analysis

**DOI:** 10.3390/brainsci12111454

**Published:** 2022-10-27

**Authors:** Hanqing Xu, Xinyan Cheng, Ting Wang, Shufen Wu, Yongqi Xiong

**Affiliations:** 1 College of Science and Technology, Ningbo University, Cixi 315211, China; 2 Department of Sociology, McMaster University, Hamilton, ON L8S 4L8, Canada; 3 Ningbo Childhood Education College, Ningbo 315336, China

**Keywords:** neuroscience, education, bibliometric analysis, research topics, research trends

## Abstract

This study aimed to explore the core knowledge topics and future research trends in neuroscience in the field of education (NIE). In this study, we have explored the diffusion of neuroscience and different neuroscience methods (e.g., electroencephalography, functional magnetic resonance imaging, eye tracking) through and within education fields. A total of 549 existing scholarly articles and 25,886 references on neuroscience in the field of education (NIE) from the Web of Science Core Collection databases were examined during the following two periods: 1995–2013 and 2014–2022. The science mapping software Vosviewer and Bibliometrix were employed for data analysis and visualization of relevant literature. Furthermore, performance analysis, collaboration network analysis, co-citation network analysis, and strategic diagram analysis were conducted to systematically sort out the core knowledge in NIE. The results showed that children and cognitive neuroscience, students and medical education, emotion and empathy, and education and brain are the core intellectual themes of current research in NIE. Curriculum reform and children’s skill development have remained central research issues in NIE, and several topics on pediatric research are emerging. The core intellectual themes of NIE revealed in this study can help scholars to better understand NIE, save research time, and explore a new research question. To the best of our knowledge, this study is one of the earliest documents to outline the NIE core intellectual themes and identify the research opportunities emerging in the field.

## 1. Introduction

Neuroscience is an important multidisciplinary scientific field in the 21st century, and its development has affected many other disciplines, including education. Interest in education, with many universities offering educational courses on neuroscience, and the use of neuroscience methods to study education-related issues are growing [1,2]. It has strongly enticed the further development of the field of education, while creating new challenges, as it provides new characterizations of the current state of the learner, including brain, genetic, and hormonal states.

The research on neuroscience in the field of education can be traced back to the 1970s [3]. However, the use of neuroscience technology, such as functional magnetic resonance imaging (fMRI), is costly and time-consuming, and only a few educators are aware of its application in the field and utilize it in their daily research work [4]. Unfortunately, this hinders the rapid development of neuroscience in the field of education (NIE). However, during the 21st century, with the birth of books such as The Social Neuroscience of Education: Optimizing Attachment and Learning in the Classroom [5], the connection between neuroscience and education has strengthened. The research on NIE has begun to attract extensive academic attention and interest. Several recent studies have reviewed the use of neuroscience for educational purposes. Sullivan et al. [6] reviewed the research literature on the neural bases of the early core deficits in autism spectrum disorder (ASD). Arantes et al. [7] reviewed the research literature on neuroanatomy teaching tools and their impact on learning. Wang [8] reviewed the relevant literature on the neural mechanisms of the constructs relevant to educational leadership, aiming to bridge neuroscience and educational leadership. Strohmaier et al. [9] reviewed the use of eye tracking in mathematics education research. Furthermore, in a systematic review, Ozel et al. [10] explored ways to use neuroimaging technology to study students’ cognitive processes and related variables in multimedia learning. Table 1 summarizes a recent review of NIE. The abovementioned reviews on NIE focus on specific fields of education, including neuroanatomy. Currently, no comprehensive review of NIE exists; many other reviews used meta-analysis. However, the sample data analyzed by meta-analysis and hand-coding were small (37-214), and hand-coding depends on the supervisor level of the researcher. Thus, the time-consuming and labor-intensive coding process may be inaccurate. Therefore, a method that can handle large-scale quantitative datasets, address the abovementioned limitations, and present the core research themes and future trends of NIE is necessary.

A bibliometric analysis is considered a very effective technique for capturing academic development trends in a research field [12]. In particular, it can be used to clarify the current state of research in a certain field and to predict future development trends [13]. Moreover, it has been widely used in many disciplines to reveal core research themes and future development trends [14,15,16,17,18,19,20].

Exploring the core knowledge in NIE is crucial; however, to the best of our knowledge, there is no effective research that examines the research achievements in NIE through relevant literature. To effectively guide the research direction of NIE, scientifically identifying the core research themes in the field, describing how the themes have evolved over time, and identifying key contributors as well as future ideas worth investing in, this study used bibliometric methods to comprehensively reveal the NIE using the science mapping software Vosviewer [21] and Bibliometrix [22] for data processing and visualization. The advantage of this study over others is that bibliometric analysis was employed to conduct a comprehensive review and overcome the author’s bias [23].

Therefore, this study performed a bibliometric analysis of 549 articles in NIE from 1995 to 2022. The most influential authors, institutions, and countries in the field, as well as the characteristics of scientific collaborations among these major contributors, are systematically introduced. The core research topics in the field and trends over time were analyzed and described. Specifically, this study makes the following four contributions: (1) It attempts to reflect the basic statistical characteristics of NIE, including the annual publication of literature, the most influential authors, institutions, countries, and highly cited literature and journals. (2) Furthermore, it describes the characteristics of the collaboration among the major contributors in NIE so that relevant researchers can find research partners. (3) It discusses the composition of basic knowledge in NIE using co-citation network analysis (4). Finally, through strategic diagram analysis and research trend analysis, the study explores the main research themes in NIE and how they evolved over time, along with future research trends. The core intellectual themes of NIE revealed in this study can help scholars to better understand NIE, save research time, and explore new research questions.

## 2. Data and Methods

### 2.1. Data Collection

The data collection comprises two parts. The first step is selecting a reliable data source. For this review, the Web of Science (WOS) core collection was selected as the data source because it can provide high-quality literature datasets and is often used in scientific research work [24]. The second step is constructing a retrieval formula. The focus of this research is on the application of NIE, so the retrieval formula constructed is TS = (Neuroscience) AND WC = (Education & Educational Research OR Education, Scientific Disciplines OR Education, Special OR Psychology, Educational), retrieval time range: 1 January 1995–30 June 2022 (cutoff date), the language type is English, the search document type is circle, and the final number of relevant documents is 549. The relevant data are presented in Table 2.

NIE has produced a total of 549 scientific papers in 27 years (1995–2022), published in 171 different journals, with an average citation of 11.38, which indicates that NIE is a research field that the academic community is more concerned about. Once the research articles in related fields are successfully published, they can arouse the interest and citations of many scholars. Simultaneously, a total of 2451 keywords, also constituting the intellectual structure of NIE, have been generated in this field. Additionally, we observed that among the 549 articles, only 132 articles were completed by one author alone, accounting for only 24%. The other 76% of scientific and technological documents were produced through collaboration, and 16.76% of which were international collaboration. These data suggest that research collaboration may be the main mode of future research output in NIE. 

### 2.2. Data Processing

The data processing process is divided into three parts.

First, for keyword merging, we combined author keywords and keywords Plus in an article. This merger method has been verified by scholars [25]. The reason for this is that many papers do not contain author keywords, which will lead to errors in subsequent research. Therefore, the keywords Plus assigned by the system are supplemented and can effectively compensate for this deficiency.

Second, we performed keyword cleaning from six aspects according to the cleaning rules proposed by [26]. Keyword cleaning included the following steps: deleting keywords without analytical meaning such as “analysis”; deleting hyphens such as that in “self-regulation”; merging singular and plural numbers such as “executive function” and “executive functions”, respectively; merging synonyms such as “young children” and “early children”; merging acronyms such as “artificial intelligence (AI)” and “functional magnetic resonance imaging (fMRI)”; and correcting spelling errors.

**Figure 1:**
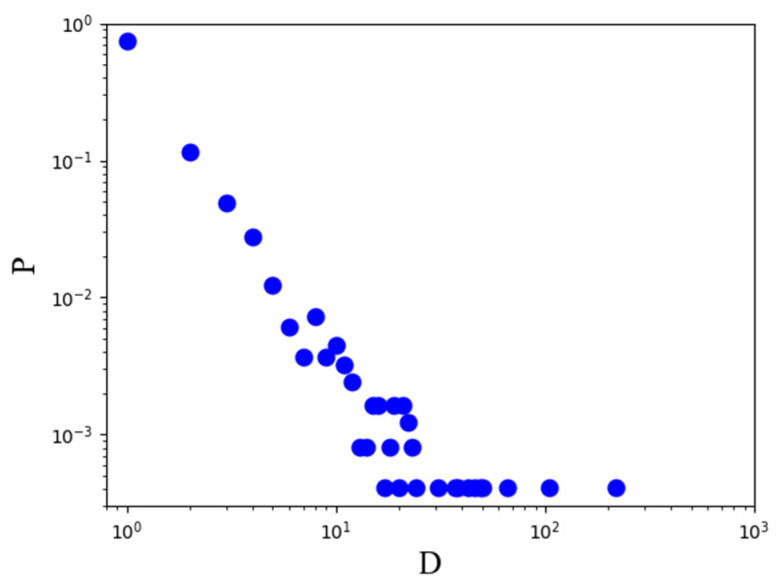
Keyword frequency distribution in neuroscience in the field of education.

Third, for keyword frequency extraction, we calculated the frequency distribution of the processed keywords, with the frequency k of the node as the abscissa, the frequency distribution probability P(k) as the ordinate, and the base 10 to establish a double logarithmic rectangular coordinate system (Figure 1). The power exponent γ of the keyword frequency distribution of NIE was approximately equal to 2.15 by power exponent fitting. According to the network scientific research results of the famous scholars Barabasi and Albert (1999) in the journal “Science”, the common structural characteristics of many large and complex networks are: the degree value of a node follows a power-law distribution, that is, the exponent γ is between 2 and 4. This indicates that the frequency of keywords in NIE obeys the power-law distribution characteristics. Thus, few keywords with high frequency act as the core in this field, representing the main research direction and main intellectual structure of this field. The same scale-free feature also demonstrates that the core knowledge topics of NIE can be characterized by few popular keywords with high frequency. Here, we extracted the keyword nodes with a frequency of >6 for subsequent analysis because the frequency of keywords reflects the research topics that scholars pay attention to. A higher frequency of keywords indicates the importance in NIE, so researchers pay more attention and interest in these high-frequency keywords. Meanwhile, the keyword frequency was >6, 112 keywords cover 93.26% (512) of the literature collection, and 101 keywords with a word frequency > 7 cover 88.89% (488) of the documents. Thus, few core keywords (frequency ≥ 6) can be used and can be more comprehensive (521 documents) to describe NIE.

### 2.3. Strategic Diagram Construction

Keyword networks are often used to analyze research knowledge topics and hotspots in disciplines [27,28]. A keyword network analysis is very intuitive and easy to interpret, and readers can read it as long as they know the keywords. First, we constructed a keyword network, the principle of which is based on the fact that two keywords synchronously appear in the same article. The Louvain algorithm [29] is often used to identify core knowledge topics for NIE, and finally, we combined keyword network, topic clustering, and network analysis (centrality and density) to form a strategy graph to characterize the knowledge structure characteristics of a research field. For each knowledge topic, we calculated its density (the topic’s internal cohesion) and centrality (how “central” the topic is to the entire field), mapping them to a strategy map to determine which of the four different states a subject can be in Figures 7 and 8. 

The first quadrant (QI) is the most mainstream knowledge topic with strong centrality and high density. The second quadrant, Quadrant II (QII), contains subjects that are well-structured internally but have insignificant external connections, with low centrality and high-density characteristics. The third quadrant (QIII) includes topics of low density and low centrality, representing emerging or disappearing research topics. Finally, the fourth quadrant (QIV) covers structurally weak, underdeveloped topics with strong centrality and low density that have the potential to significantly impact the field as a whole. Based on the evolution in a time series, observing the evolution and distribution characteristics of knowledge topics is more helpful in grasping the mainstream research direction of NIE.

## 3. Results

### 3.1. Annual Publications Analysis

**Figure 2:**
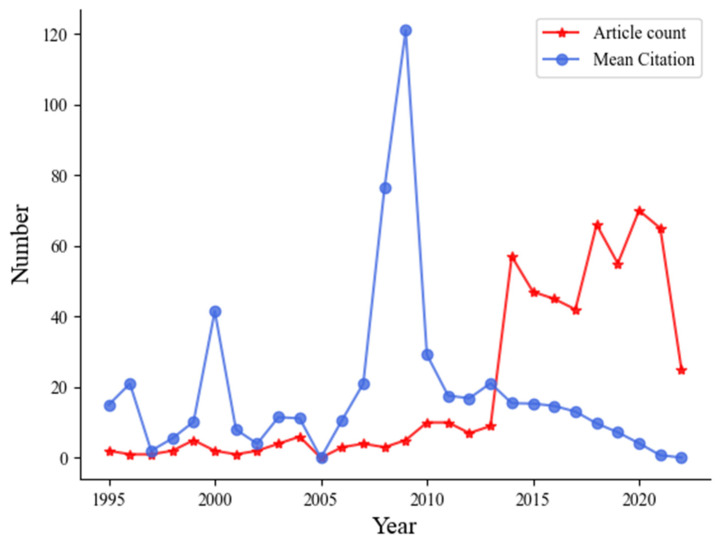
Publishing trends of neuroscience in the field of education.

Through the statistical analysis of the chronological distribution of NIE papers, grasping the theoretical level and development speed of related research is helpful. As presented in Figure 2, the growth trend of NIE papers is divided into two stages as follows: the first stage is the stable growth stage from 1995 to 2013, the research on NIE is in its infancy, and the maximum number of papers published yearly is approximately 10. Early scholar research focused on incorporating neuroscience as a core course into the university education system, and attempted to integrate it with different course teachings, while taking surveys to observe the feedback effects of neuroscience-related courses. Although early development was slow, the rise of neuroscience technology laid the groundwork for its teaching and application in education. The second stage is the explosive growth stage from 2014 to 2022. The research literature around this field was in a state of blowout. Although the number of literatures declined from 2015 to 2016, it rebounded rapidly in 2017, and the overall trend was upward. The formation of this stage is due to the continuous popularization and improvement of the teaching curriculum design in NIE, and the transfer of neuroscience from curriculum teaching to practical application has become the focus of scholars. Meanwhile, an increasing need for innovative technological methods such as “neuroscience” to solve education-related problems has been observed. Figure 2 demonstrates the rapid development of research results of NIE and the booming trend of the overall performance.

### 3.2. Performance Analysis

#### 3.2.1. The Most Influential Countries

Table 3 lists the top 10 countries for research output in NIE, where the United States is identified as the most productive and influential country. Since 1995, a total of 248 papers have been published, and a total of 3743 citations have been received. The United States followed by England and Australia with 83 papers (829 citations) and 39 papers (335 citations), respectively. These countries have made great contributions to the development of neuroscience.

#### 3.2.2. The Most Influential Institutions

A total of 677 institutions participated in the research work of NIE, and 172 institutions have published more than two papers, accounting for a quarter of the total number of institutions. This suggests that the research on NIE by many research institutions is in the trial stage, and the number of in-depth research institutions is not large. We extracted the top 10 institutions in terms of the number of published papers (Table 4). Among them, Harvard University is the institution with the largest number of papers, with a total of 15 papers published and a total of 227 citations, with an average number of citations per paper of 15.13. Subsequently, Stanford University published 12 articles, with a total of 190 citations, and the average number of citations per article was 15.83, while the most cited institution was the University of California, Los Angeles, which only published 7 articles, but received 346 citations, all of which were cited 49.43 times. Of note, six of the top 10 institutions are from the United States.

#### 3.2.3. The Most Influential Authors

We list the top 10 high-yield authors((Table 5), of which DECETY J and TRAVIS MJ both have published five articles each, and are the most productive scholars in this field. The studies of the two scholars spanned from 2014 to 2021. DECETY J’s early research focused on the impact of empathy on medical students in medical training. He stated that empathy is an important aspect of clinical care, and improving medical students’ empathy can improve patient satisfaction, increase treatment compliance, and reduce medical errors and complaints to make changes [30]. Later studies are transferred to third-party moral evaluations in children [31,32,33]. Travis has focused on the integration of neuroscience curriculum education into the clinical practice of psychiatry, aiming to develop an interdisciplinary NIE curriculum for psychiatry to help psychiatric residents better grasp the latest scientific technology and training requirements, involves changing the teaching content of neuroscience courses through the findings of the neuropsychiatry and neuroscience education of psychiatry trainees on learning attitudes and impairments [34], realizing the possibility of educating neuroscience courses across regions by combining small private online course technology with flipped classroom technology [35], and developing an interdisciplinary neuroscience curriculum based on a multi-departmental teamwork [36,37]. Drake and McBride are the most cited scholars, and the two scholars have a cooperative relationship. Their research focuses on Reform of Educational Anatomy [38,39,40].

#### 3.2.4. The Most Influential Papers

Table 6 lists the articles ranked top 10 by citation frequency to indicate the most representative literature in this field. Papers on the NIE curriculum account for six papers, ranking first (519), third (161), fifth (124), sixth (113), and seventh (112) in citations. Among them, Drake, McBride, Lachman, and Pawlina [38], in order to match the latest progress of medical education in the anatomical sciences, a questionnaire was used to investigate the changes in 130 allopathic and 25 osteopathic medical schools in the United States in neuroscience/neuroanatomy and other courses in the past 10 years from 2002 to 2009. The research results provide strong data support for the curriculum reform of related neuroscience. The article has been cited 519 times to date. This work is ongoing, and in 2014 [39] and 2018 [40], Drake RL and McBride JM launched several national surveys to discover the drivers of medical education curriculum reform and their effects on anatomy, neuroanatomy/neuroscience, and embryology courses to enable relevant curriculum reforms. This work is of forward-looking significance. With the continuous change in society, the continuous integration of new technologies and new tools, and the timely grasp of the latest innovative teaching methods, the incorporation of technical means is crucial for the development of the discipline. The fifth most cited article was published in “BMC Medical Education” in 2010. The article was based on a survey of senior studies and residents of a medical school in the United States and investigated their difficulties in dealing with patients with neurological disorders, identified the perceived sources of these difficulties, and provided recommendations for developing an effective educational experience in neurology [41]. The sixth most cited article, published in 2010 in “Anatomical Sciences Education”, introduces a three-dimensional (3D) visualization tool for neuroanatomy and evaluates the learning and use of the software by setting up a control group of students. The findings suggest that the use of 3D neuroanatomical visualization tools can better assist the development of higher education in neuroscience, neurology, and neurosurgery [42]. The other four are applications of neuroscience in education, including the second most cited article written by Epstein et al. [43], in which he stated that cognitive neuroscience can probe brain functions related to self-monitoring, which will help clinicians to improve their clinical practice methods and quality of care they provide by self-monitoring their abilities. The fourth and seventh most cited articles used neuroscience techniques to measure the efficacy of empathy training [44] and the link between intelligence and creativity [45]. Finally, the 9th and 10th most cited are review articles on synaptic plasticity [46] and educational games [47], respectively. 

#### 3.2.5. The Most Influential Journals

Table 7 lists the 10 journals with the highest number of NLE research articles published in the period 1995–2022. Respectively, Mind Brain and Education (publications = 52), Academic Psychiatry (publications = 32), Atomical Sciences Education (publications = 31), Advances in Physiology Education (publications = 29), BMC Medical Education (publications = 14), Academic Medicine (publications = 13), Educational Philosophy and Theory (publications = 13), NPJ Science of Learning (publications = 12), E-Life Sciences Education (publications = 10), and Early Child Development and Care (publications = 10). The journal of “Anatomical Sciences Education” was cited the most with 1174 citations. Of the top 10 highly cited papers, four are from the abovementioned journal (Table 6). Thus, neuroscience education research is one of the important research contents of NIE.

### 3.3. Collaboration Network Analysis

#### 3.3.1. Country Collaboration Network

**Figure 3:**
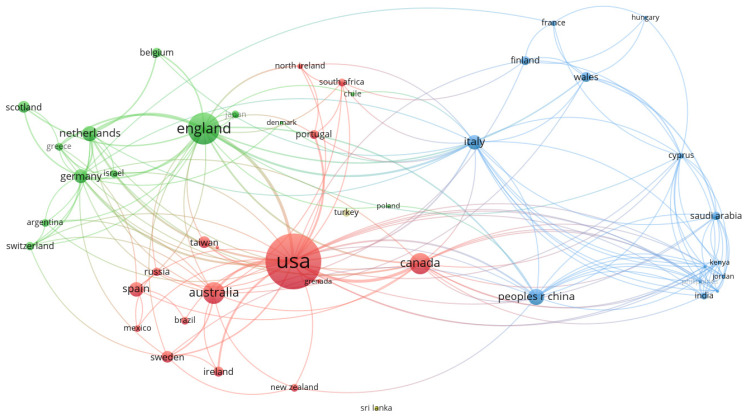
Country collaboration network.

We built a country collaboration network based on the cooperative relationship between countries. Figure 3 depicts 57 nodes and 183 edges, where one node represents a country, and a line represents a cooperative relationship between countries. The thickness of the line indicates the degree of cooperation between countries. The thicker the line, the closer the collaboration between the two countries. Among them, 45 nodes (countries) form the largest connected piece, and the United States plays an important role in the national collaboration network, with the highest degree of collaboration, and has cooperative relations with 31 other countries, followed by England, Italy, Canada, Cyprus, and China. These countries not only rank high in the number of their own publications, but also occupy a core position in the national collaboration network. Thus, international collaboration is a trend in the future, which can positively impact scientific research output.

#### 3.3.2. Institutional Collaboration Network

**Figure 4:**
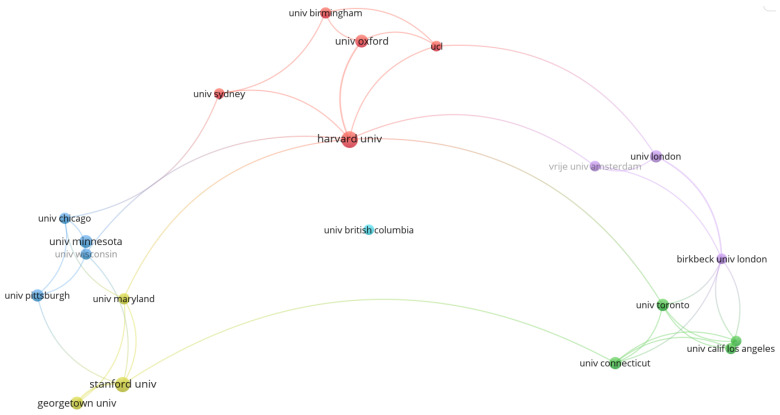
Institutional collaboration network.

Figure 4 provides the collaboration network of institutions with over 6 times publication frequency, including a total of 20 nodes (institutions) and 60 connections, including Harvard University, Stanford University, University of Maryland, University of Wisconsin, University of Pittsburgh, University of Chicago, and Birkbeck University. Harvard University has the highest degree of collaboration, and these institutions have cooperated with other 10, 8, 7, 6, 6, 6, and 6 institutions at least once. Meanwhile, the vast majority of institutions in the network are from the United States, with close collaboration between American institutions. The institutions with the largest number of papers published through collaboration are Harvard University (11) and Stanford University (11), followed by the University of Maryland (10). 

#### 3.3.3. Author Collaboration Network

**Figure 5:**
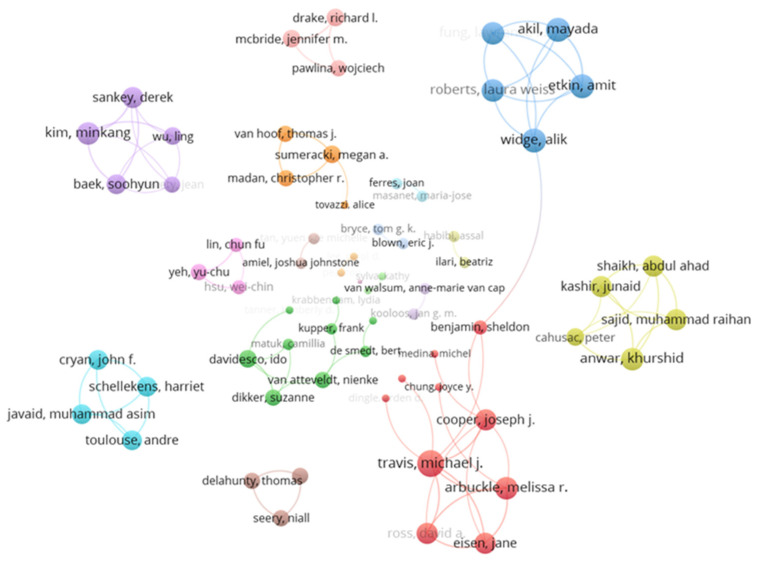
Author collaboration network.

Figure 5 presents the author collaboration network with over two published articles, a total of 93 authors and 83 links, in which the nodes represent the authors, and the links represent the cooperative relationship between the authors. Several cooperative groups are formed among different authors, and the largest subgroup contains 15 authors. Conducting neuroscience education work is also a core research content of the group’s branch.

### 3.4. Co-Citation Network Analysis

**Figure 6:**
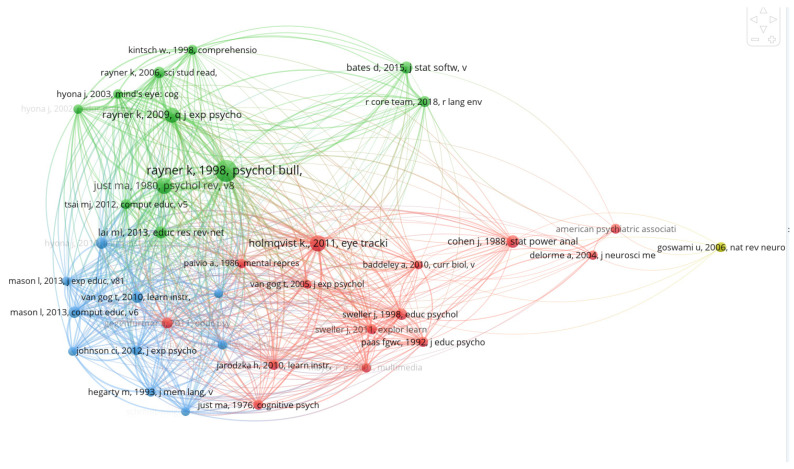
Co-citation network.

If two papers (or papers) are simultaneously cited in one or subsequent papers, the two papers constitute a co-citation relationship. We constructed a co-citation network based on the co-citation relationship between 25,886 references from 549 articles to identify those representative classic documents in NIE. We set the threshold citation frequency ≥ 30 for network-level extraction, a total of 37 of the most representative works, including 6 books [48,49,50,51,52,53] and 31 papers, using a clustering algorithm to form a total of 4 knowledge communities, as shown in Figure 6. Due to the limited length of the paper, we will select the most representative three documents from each knowledge community for content description.

#1 (Yellow): This knowledge community focuses on neuroscience and education. Goswami [54] stated that teachers are currently at the receiving end of numerous “brain-based learning” packages. Some of these contain alarming amounts of misinformation, yet such packages are being used in many schools. Therefore, the question is how neuroscientists can transfer relevant professional knowledge in a more digestible way to bridge the gap between neuroscience and education. This is important for educators and education departments. Howard-Jones et al. [55] demonstrated that the differences in terminology and language created a gap between neuroscience and education. Thus, in schools and universities, neuromyths are often used to prove the appropriateness of ineffective teaching methods and distort scientific facts. This is because there exists a gap in the communication between teachers and neuroscientists. Therefore, it is of the utmost importance to establish a new research field connecting neuroscience and education to inform and improve the NIE application.

#2 (Red): This knowledge community focuses on theory and methodology. Thus, Just [56] proposed a reading comprehension model, which embedded a theoretical framework that can adapt to the flexibility of reading. The content of the theoretical framework specifically includes the following three aspects: reading people, reading goals, and reading content. This model can effectively explain the starting duration of college students pertaining to reading scientific articles. Furthermore, Sweller et al. [57] designed cognitive load theory, which aims to help educators design various novel teaching procedures and provide guidelines intended to assist in the presentation of information in a manner that encourages learner activities that optimize intellectual performance. Holmqvist et al. [53] published a book titled Eye Tracking: A Comprehensive Guide to Methods and Measures to help relevant researchers better understand and use eye movement tracking technology to complete relevant research work. The book has been cited 3811 times in Google Academic.

#3 (Green): This knowledge community focuses on literature review. Rayner et al. [58] reviewed the relevant literature on eye movements in reading and other information-processing tasks. Basic topics discussed with respect to reading were the characteristics of eye movements, the perceptual span, and integration of information across saccades, eye movement control, and individual differences (including dyslexia). Then, in 2006, Rayner et al. [59] again reviewed the relevant literature on basic features of eye movement during reading, discussing the possibility of using eye movement data to evaluate the instantaneous understanding process in a school environment. Lai et al. [60] reviewed the eye-tracking technology in exploring learning based on 81 papers from the Social Sciences Citation Index database. The research conclusion shows that the eye-tracking method provides a suitable way to link learning results with cognitive processes.

#4 (Blue): This knowledge community focuses on multimedia learning. Hannus et al. [61] studied through two experiments the influence of illustrations on the learning curriculum materials of 10-year-old pupils with high and low intelligence. The research results show that the appearance of illustrations improves the learning of illustrations text content. Students with higher intelligence are easier to learn illustrations in science textbooks, and their comprehension scores can be improved due to the appearance of illustrations. Mason et al. [62] used eye-tracking technology to measure three dimensions of free recall, factual knowledge, and knowledge transfer to detect the online processing of text and graphics by fourth graders when reading illustrations science texts. In 2013, Mason et al. [63] again investigated the online process of reading and offline learning from an illustrated science text based on 59 eleventh-grade students through eye tracking. Results showed that the text illustrated by either the concrete or the abstract picture led to better learning than text alone.

### 3.5. Research Themes Analysis

#### 3.5.1. Core Research Themes for 1995–2013

**Figure 7:**
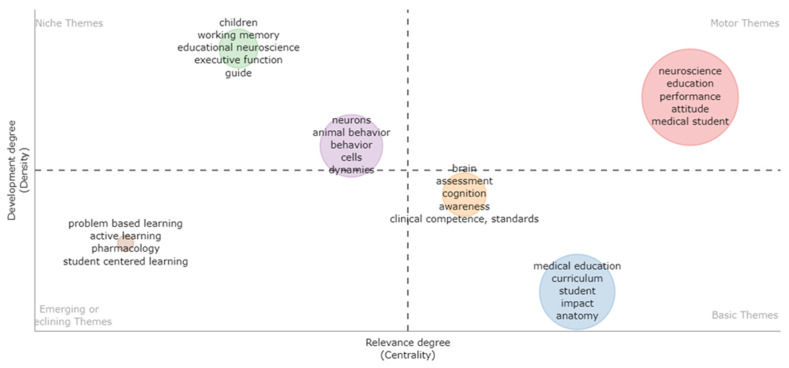
Strategic diagram for neuroscience in the field of education for 1995–2013.

To study the paradigm change in NIE over the last 27 years (1995–2022), we divided the data into two parts according to the development cycle of the field: 1995–2013 and 2014–2022. From 1995 to 2013, due to the small number of keywords, we extracted nodes with keyword frequency ≥ 2, a total of 58 keyword nodes, and obtained five knowledge communities using the Louvain algorithm. Each knowledge community represents a research subfield or research topic in NIE. The relevant results are presented in Table 8 and Figure 7.

As depicted in Figure 6, NIE is a popular research knowledge topic C01 (neuroscience, education, performance, attitude, and medical student) located in the QI of the strategic diagram. This knowledge community revolves around assessing the performance of students when neuroscience education introduces new school tools and technologies. For example, Brueckner and Traurig [64] investigated student acceptance of a digital laboratory guide in a medical neuroscience course. Goldberg and McKhann [65] evaluated the effectiveness of delivering the core curriculum of an introductory neuroscience course using a virtual learning interface. Software tools, such as a lecture recording system [66], online multimedia teaching tool [67], and BrainExplorer [68], were used to investigate the performance of assessments on student learning and achievement.

The QII of the strategic diagram contains two knowledge communities. The first is C02 (children, working memory, educational neuroscience, and executive), which covers the use of neuroscience for observing the impact of educational games on student outcomes and learning experiences [69,70], and brain memory function in children with intellectual disabilities [71]. The second is C03 (neurons, animal behavior, behavior, cells, and dynamics), in which research scholars on this subject of knowledge mainly focus on the relevant research and experimental work in the teaching process using neuroscience [72,73,74]. The QIII of the strategic diagram contains one knowledge community named C4 (problem-based learning (PBL), active learning, pharmacology, and student-centered learning). This knowledge topic has only been covered by three studies, which mainly compared the academic performance of students who underwent a traditional curriculum versus those with a PBL curriculum [75,76].

The QIV of the strategic diagram contains two knowledge communities. The first is C5 (brain, assessment, cognition, awareness, clinical competence, and standards), in which scholars focus on using neuroscience technology to conduct self-assessment of clinicians to enhance and improve their level of professional knowledge [43,77]. The second is C6 (medical education, curriculum, student, impact, and anatomy), in which neuroscience education is integrated into the curriculum of medical teaching. This is also the main research work of NIE in the early days. Rubin and Zorumski [78] have reported that neuroscience affects the diagnosis and treatment of psychiatry, and this influence will grow significantly in the future. Hence, neuroscience should be integrated into undergraduate medical education to help psychiatry departments develop the necessary leadership and expertise. Ruiter et al. [79] elaborated that a major challenge in contemporary research is how to connect medical education and cognitive neuroscience and realize synergies between these fields. Solving this problem is crucial for the further development of medical education. Some scholars have also conducted more fine-grained research on the medical education of undergraduates [80] and graduate students [81] integrating neuroscience courses.

#### 3.5.2. Core Research Themes for 2014–2022

From 2014 to 2022, a total of 475 documents were produced. We extracted 112 keywords with a keyword frequency ≥ 6 times. Using the clustering algorithm, four knowledge communities were formed in Table 9 and Figure 7.

**Figure 8:**
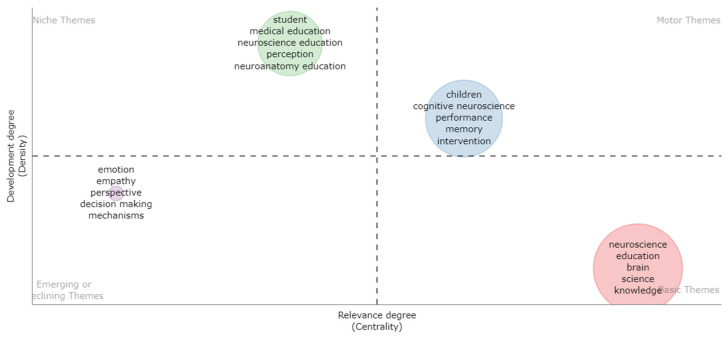
Strategic diagram for neuroscience in the field of education for 2014–2022.

As presented in Figure 8, the QI of the strategic diagram contains one knowledge community: C7 (children, cognitive neuroscience, performance, memory, and intervention). The C7 is the most popular research topic and the largest knowledge community in 2014–2022. It contains a total of 39 core keywords and 169 articles. This topic mainly uses neuroscience technology to investigate children’s cognitive function for their healthier development. For example, Sullivan, Stone, and Dawson [6] reviewed the research literature on the neural bases of the early core deficits in ASD and proposed three key features of early intervention related to the neural mechanisms that may contribute to its effectiveness in improving deficit areas. Berninger et al. [82] used the FMRI technology to measure the brain activity of students with specific learning disabilities (SLDs). Their results showed differences in neuroimaging patterns and brain region connections between students with dysgraphia, dyslexia, and oral and written language learning disability (OWLLD) and normal students. Finally, this group of authors stated that transforming this research into educational practice required changing policies and government regulatory procedures. Dias et al. [83] proved that teachers’ intervention in students’ executive function(EF) in the classroom could improve students’ reading and arithmetic performance. Therefore, EF intervention could be a useful tool to improve adjustment and academic achievement. Zhang et al. [84] developed an intervention combining reading training and transcranial direct current stimulation (tDCS) to improve orthographic awareness and reading fluency of children with reading disabilities. The results indicate that the combination of literacy teaching and tDCS plays effectively improves reading abilities in children with reading disabilities. Howard et al. [85] used functional magnetic resonance imaging to investigate the impact of assessment methods on neural processing and performance in young children.

The QII of the strategic diagram contains one knowledge community: C8 (student, medical education, neuroscience education, perception, and neuroanatomy education). Curriculum reform related to neuroscience is the focus of research in this knowledge community. In C8, new software tools are incorporated [86,87] and the curriculum undergoes cross-disciplinary reform [88,89]. 

The QIII of the strategic diagram contains one knowledge community: C9 (emotion, empathy, perspective, decision making, mechanisms, and social neuroscience). The C9 is a new knowledge topic that emerged in 2016. Since the first article on this topic was published, the literature on this topic has received extensive attention and citations in the academic community. This topic of knowledge examines the efficacy of empathy in different domains and specific populations, such as empathy in social work [90], empathy in medicine [91], empathy for patients [92], and empathy for vulnerable groups [93].

The QIV of the strategic diagram contains one knowledge community: C10 (neuroscience, education, brains, science, and knowledge). This knowledge topic focuses on understanding brain development and learning. More specifically, it can be divided into three aspects: (1) teachers’ cognition of the brain and education; for example, Gleichgerrcht et al. [94] assess 3451 Latin American teachers’ beliefs regarding neuroscience, especially when it comes to factual information on its structure and function. Dundar and Gunduz [95] used a questionnaire to investigate misconceptions regarding brain and neuroscience among pre-service teachers. Tovazzi et al. [96] conducted the most commonly used and new questionnaires among 174 Italian teachers to collect the characteristics of teachers’ attachment and belief in neuroscience. It should be noted that this topic of knowledge is not the mainstream research content of NIE. (2) brain structure and activity with learning. For example, according to Wenger et al. [97], the hippocampus is a highly plastic brain structure and is important for learning and memory. The volume of the hippocampal itself may indicate the success of learning, such as the acquisition of arithmetic skills, which implies that it can be used for new measures of the effects of learning and teaching. Furthermore, Lee et al. [98] pointed out that previous studies have proved that the lateral prefrontal cortex (LIPFC), horizontal parietal sulcus (HIPS), and fusiform area and angular gyrus play an important role in solving mathematical problems. In this study, fMRI was used to observe the effects of mathematical teaching types (oral and example) on brain activity. The results show that LIPFC is related to mathematical cognition, and HIPS plays a role in mathematical tasks and computing processes. In the example teaching process, activities in the forehead area, including LIPFC, and parietal lobe area, including HIPS, were increasing; however, the oral teaching process had a number of LIPFC activities. The research results provide support for when example-based teaching is more appropriate. Borst et al. [99] said that reading depends on the left-side network of the brain region, which has long been accepted by the academic community. However, there is little research on the influence of the brain of school-age children on reading skills. Therefore, this study used anatomical MRI to study the influence of the sulcus pattern of lateral OTS on oral reading skills. Our findings indicate that the left OTS sulcus pattern affects reading skills. This study provides an effective way for teachers to identify children at risk of developing poor reading skills. Luk et al. [100] provided an overview of brain structural (diffusion tensor imaging, DTI; and voxel-based methodology, VBM) and functional methods in the process of second language learning. (3) Theory, method, measures of brain and education. For example, Liu et al. [101] proposed six concept learning constructs based on brain theory, including understanding brain function; utilizing mind maps, mnemonics, and other learning devices; building pattern recognition and consciousness; building concrete multisensory experience; injecting infuse multimedia into the educational environment; and infusing positive emotions into the educational environment. This theory is valuable for results-based education. Van et al. [102] proposed a framework called Responsible Research and Innovation to improve the alignment between mind, brain, and education research. Dahlstrom et al. [103] said the traditional educational evaluation methods could not evaluate students’ implicit learning and proposed the use of multimodal neurocognitive tools, such as EEG, fNIRS, and eye tracking, to measure implicit learning in the classroom. They demonstrated the potential of using neurocognitive tools to provide measures beyond those commonly available in traditional education assessment from the following three aspects: attention, working memory and cognitive load, and long-term memory.

#### 3.5.3. Evolution Analysis of Core Research Themes

Compared with the first stage (1995–2013), the number of core keywords in the second stage (2014–2022) has greatly increased, while the core knowledge community has decreased. This is due to the initial research in NIE being very random, and scholars will attempt all possible research directions. With the continuous development and accumulation of academic achievements in NIE, the number of core knowledge communities in the second phase of research is reduced. We identified that the cross-integration of core knowledge topics C1 and C5 evolved in C10, a knowledge topic with a weak network structure. Meanwhile, knowledge communities C2 and C3 started declining. Scholars have rarely engaged in research work on these two knowledge topics, and knowledge topic C2 has evolved into C7, thus becoming the most popular core knowledge community in the second stage. The knowledge community C9 emerges as a new knowledge topic.

### 3.6. Research Trend Analysis

**Figure 9:**
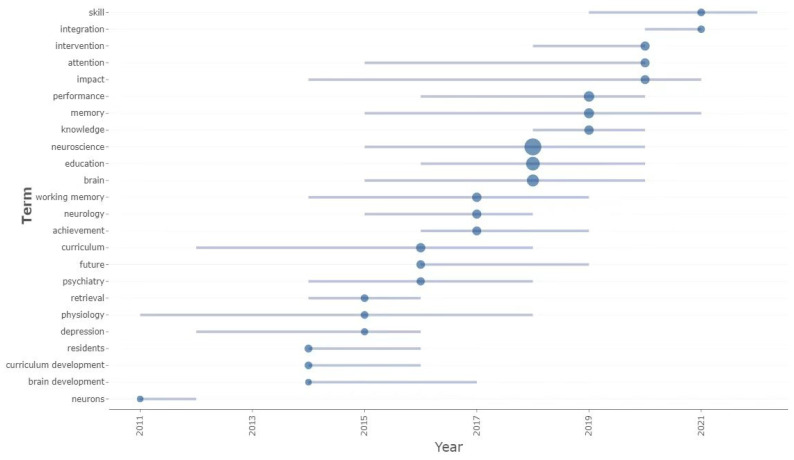
Theme evolution trend.

We used the year as the time window to describe the evolution trend of core keywords. As presented in Figure 9, the most mentioned keywords in the past 3 years are skill and integration. The related research around the keyword “skill” is the use of neuroscience technology to improve the educational process of children in order to improve writing [104], number recognition [105], accessible reading [106], and other skills. Related research around the keyword “integration” involves the design and reform of neuroscience courses for medical students. Balta et al. [107] present the universal design for a learning framework, an informed, evidence-based, and robust approach to underpin new course design and pedagogical reform in anatomy education. Porter-Stransky and Gallimore [108] explored neuroscience and psychiatry integration in undergraduate medical education, followed by core keywords such as “intervention”, “attention”, “impact”, and “performance”. The main research content involved exploring the impact of integrating new learning models [109] and new technologies [110] in neuroscience education in students. Expectedly, children as the research object will be the mainstream of future research in NIE. Thus, how to use neuroscience technology in order to help relevant educators in improving children’s education, cognition, skills, and learning ability is a future research trend. Moreover, the related changes and evaluation of neuroscience courses remain the subject of future research in NIE.

## 4. Conclusions and Discussion

### 4.1. Research Conclusions

The research work is based on the literature of NIE included in the Web of Science Core Collection from 1995 to 2022. Using a bibliometric analysis, the core authors, institutions, countries, articles, and journals of the research in NIE were identified. The development of core knowledge topics over time is described. Based on the results, the research work initially draws the following conclusions:

First, the performance analysis revealed that the most influential country is the United States. that Harvard University from the United States is the most influential research institution, that Decety and Travis are the most influential scholars, and that Anatomical Sciences Education is the most influential journal. This information can help researchers working in the field, such as selecting the right partner and right journal to submit their manuscript. 

Second, through the strategic diagram, we discovered that children and cognitive neuroscience, students and medical education, emotion and empathy, education, and the brain are core intellectual themes of current NIE research. Exploring the cognitive characteristics of children’s brains can help in conducting educational work better and help children develop better academically. Moreover, skills remain the focus of most scholars’ attention [111,112]. Discussing neuroscience curriculum reform for medical students has become a highly specialized issue, although the subject of research in NIE is not very popular. Emotion and empathy are emerging themes in NIE. In particular, examining the efficacy of empathy in children [113,114] is a research direction that has emerged in recent years. Understanding brain development and education is a fundamental research topic in NIE, but the number of citations for papers published on this topic in recent years shows that it is becoming less attractive to scholars.

### 4.2. Implications for Academic Research

The application of neuroscience techniques in education is not perfect. An important reason is that neuroscience methods are often considered incompatible with educational theory and limit its development in teaching [115]. NIE is more than simply integrating neuroscience into education; it is an interdisciplinary field. To address the theoretical, methodological, and technical challenges faced by researchers in education when using neuroscience techniques, interdisciplinary collaboration is necessary. Studies have demonstrated that interdisciplinary research areas have a greater long-term impact than single-disciplinary research [116]. Therefore, interdisciplinary cooperation should also be considered a future research direction in NIE.

NIE is considered a subject area that connects education and neuroscience. However, difficulties in data quality and data analysis limit its development in education, and many educators are unable to determine whether the neuroscience technique of choice is suitable for a particular study and what insights will be added through it. In response to this issue, Parada and Rossi [117] highlighted the importance of theoretical and methodological needs for the use of neuroscience-related devices in their published article. Medina et al. [118] surveyed the need for neuroimaging education among psychiatry residents at seven U.S. universities, and the data revealed that psychiatrists have a strong interest in neuroimaging education. Future educational interventions should address this need. Therefore, developing education and training for scholars in the field of pedagogy using neuroscience techniques will be one of the focuses of future research.

Existing neuroscience research has confirmed that the left brain is responsible for language, logic, mathematics, analysis, and other thinking information processing, while the right brain is responsible for processing visual information, such as space, images, and other information processing. When neuroscience technology enters the classroom, it connects the brain with learning, which brings new insights into the development of cognitive function. For example, through the study of brain function, we can distinguish automatic learning from rote learning [119]. It can not only help teachers improve teaching strategies effectively [120] but also evaluate students’ learning outcomes [121]. It is foreseeable that the application of neuroscience in learning and teaching is the future trend in education. However, cognitive neuroscience research is usually conducted in a controlled laboratory environment, and thus its contribution to our understanding of learning in the real world is limited. Therefore, future research should consider the development of portable devices so that neuroscience research can truly enter the classroom and enable real-time assessment of teaching and learning [122].

### 4.3. Research Limitations

This study provides a comprehensive review of NIE using bibliometric analysis to help the NIE develop better. However, this study had some limitations. First, this study only adopted journal papers from the core set of WOS, and some research in the field of NIE was published in conference papers or books, so they were not within the scope of our analysis, which may bring errors to our research results. Therefore, future research could adopt more databases, such as SCOPUS and EBSCO. Another limitation is how well an author’s keywords are prepared to reflect a paper. Authors rely entirely on their habits and cognition when assigning keywords to the paper they write. This may result in an error. Therefore, in the future, the structural topic model [123] needs to be considered when extracting topic words from text information such as abstracts and titles of papers.

## Figures and Tables

**Table 1:** A recent review of neuroscience in the field of education.

Author	Research Topic	Number of Reviewed Articles
[7]	Tools and resources for neuroanatomy education	214
[8]	Neuroscience and educational leadership	73
[9]	Eye-tracking methodology in mathematics education	161
[10]	Neuroimaging tools in multimedia learning	40
[11]	Brain-imaging techniques in educational technologies	37

**Table 2:** Basic statistical description.

Description	Results
Timespan	1995:2022
Journals	171
Documents	549
Annual growth rate (%)	9.81
Average citations per document	11.38
References	25,681
Keywords	2451
Authors	1584
Authors of single-authored documents	132
Co-authors per document	3.14
International co-authorships (%)	16.76

**Table 3:** Top 10 most influential countries.

Rank	Country	Papers	Citations
1	USA	248	3743
2	England	83	829
3	Australia	39	335
4	Canada	37	325
5	People’s Republic of China	22	121
6	Netherlands	19	215
7	Spain	18	90
8	Italy	17	169
9	Germany	16	95
10	Sweden	12	102

**Table 4:** Top 10 most influential institutions.

Rank	Institution	Papers	Citations
1	Harvard University	15	227
2	Stanford University	12	190
3	Georgetown University	9	70
4	University of Oxford	9	94
5	University of Pittsburgh	8	113
6	University of Connecticut	8	120
7	University of Toronto	8	158
8	University of London	8	127
9	University of Minnesota	8	54
10	University of California, Los Angeles	7	346

**Table 5:** Top 10 most influential authors.

Rank	Author	h_index	g_index	TC	NP	PY_start
1	Decety J	4	4	119	5	2014
2	Travis MJ	4	4	46	5	2014
3	Akil M	3	3	30	3	2014
4	Benjamin S	3	3	45	3	2014
5	Cooper JJ	3	3	33	3	2014
6	Drake RL	3	3	755	3	2009
7	Etkin A	3	4	68	4	2014
8	Lee CD	3	3	31	3	2016
9	Mcbride JM	3	3	755	3	2009
10	Ross DA	3	3	34	3	2014

**Table 6:** Top 10 most influential papers.

Author	Title	Journal	Citation
Drake (2009) [38]	Medical education in the anatomical sciences: the winds of change continue to blow	Anatomical Sciences Education	519
Epstein (2008) [43]	Self-monitoring in clinical practice: a challenge for medical educators	Journal of Continuing Education in the Health Professions	185
Drake (2014) [39]	An update on the status of anatomical sciences Education in United States medical schools	Anatomical Sciences Education	161
Van Berkhout and Malouff (2016) [44]	The efficacy of empathy training: a meta-analysis of randomized controlled trials	Journal of Counseling Psychology	138
Estevez (2010) [42]	A novel three-dimensional tool for teaching human neuroanatomy	BMC Medical Education	114
Zinchuk (2010) [41]	Attitudes of us medical trainees toward neurology education: “neurophobia”, a global issue	Anatomical Sciences Education	113
Mcbride (2018) [40]	National survey on anatomical sciences in medical education	Anatomical Sciences Education	112
Silvia (2015) [45]	Intelligence and creativity are pretty similar after all	Educational Psychology Review	99
Abraham (2019) [46]	Is plasticity of synapses the mechanism of long-term memory storage?	NPJ Science of Learning	92
De freitas (2018) [47]	Are games effective learning tools? A review of educational games	Educational Technology & Society	77

**Table 7:** Top 10 most influential journals.

Rank	Sources	Articles	Citations
1	Mind Brain and Education	52	374
2	Academic Psychiatry	32	241
3	Anatomical Sciences Education	31	1174
4	Advances in Physiology Education	29	237
5	BMC Medical Education	14	272
6	Academic Medicine	13	363
7	Educational Philosophy and Theory	13	37
8	NPJ Science of Learning	12	162
9	CBE-Life Sciences Education	10	116
10	Early Child Development and Care	10	25

**Table 8:** Major research themes in neuroscience in the field of education for 2003–2018.

ID	Q	Most Frequently Used Keywords	Size	Total Frequency
C01	QI: motor themes	Neuroscience (20); education (13); performance (7); attitude (5); medical student (4)	19	84
C02	QII: niche themes	Children (5); working memory (3); educational neuroscience (2); executive function (2); guide (2)	6	16
C03	QII: niche themes	Neurons (5); animal behavior (3); behavior (3); cells (2); dynamics (2)	12	29
C04	QIII: emerging or declining themes	Problem-based learning (3); active learning (2); pharmacology (2); student-centered learning (2)	4	9
C05	QVI: basic themes	Brain (7); assessment (4); cognition (3); awareness (3); clinical competence standards (2)	7	22
C06	QVI: basic themes	Medical education (11); curriculum (11); student (9); impact (6); anatomy (5)	10	40

**Table 9:** Major research themes in neuroscience in the field of education for 2014–2022.

ID	Q	Most Frequently Used Keywords	Size	Total Frequency
C07	QI: motor themes	Children (50); cognitive neuroscience (40); performance (35); memory (30); intervention (23)	36	511
C08	QII: niche themes	Student (39); medical education (35); neuroscience education (24); perception (21); neuroanatomy education (20)	28	400
C09	QIII: emerging or declining themes	Emotion (20); empathy (17); perspective (11); decision making (10); mechanisms (8)	11	102
C10	QIV: basic themes	Neuroscience (201); education (96); brain (62); science (46); knowledge (24)	37	739

## Abbreviations

3D: three-dimensional; ASD, autism spectrum disorder; NIE, neuroscience in the field of education; PBL, problem-based learning.
